# Neural Signatures of Gender Differences in Interpersonal Trust

**DOI:** 10.3389/fnhum.2020.00225

**Published:** 2020-06-16

**Authors:** Yan Wu, Alisha S. M. Hall, Sebastian Siehl, Jordan Grafman, Frank Krueger

**Affiliations:** ^1^Department of Psychology, College of Education, Hangzhou Normal University, Hangzhou, China; ^2^Zhejiang Key Laboratory for Research in Assessment of Cognitive Impairments, Hangzhou Normal University, Hangzhou, China; ^3^Department of Psychology, University of Mannheim, Mannheim, Germany; ^4^Department of Cognitive and Clinical Neuroscience, Medical Faculty Mannheim, Central Institute of Mental Health, Ruprecht-Karls-University Heidelberg, Mannheim, Germany; ^5^Graduate School of Economic and Social Sciences, University of Mannheim, Mannheim, Germany; ^6^Shirley Ryan AbilityLab, Northwestern University, Chicago, IL, United States; ^7^School of Systems Biology, George Mason University, Fairfax, VA, United States; ^8^Department of Psychology, George Mason University, Fairfax, VA, United States

**Keywords:** trust game, gender, risk, parental investment theory, social role theory, subgenual anterior cingulate cortex, inferior frontal gyrus, precuneus

## Abstract

Trust plays a critical role in nearly every aspect of social life. Parental investment theory and social role theory predict that women trust less than men due to a higher sensitivity to risk and betrayal, while men trust more than women to maximize resources and to signal their willingness to lose something. However, the underlying neuropsychological underpinnings for this gender difference are still obscure. In this study, we used functional magnetic resonance imaging (fMRI) to investigate the neural signatures of gender differences in trust by simultaneously scanning 11 male and 11 female same-gender, fixed dyads who played a multi-round binary trust game with varying levels of payoff (low/moderate/high) as an indicator of social risk. Our results showed that men trusted more than women and payoff level moderated the effect of gender on trust. While men trusted the same at all payoff levels, women trusted less with higher payoff levels. This pattern was supported by our neuroimaging finding: men showed a higher activation in the left inferior frontal gyrus (ventrolateral prefrontal cortex) and right precuneus than women, indicating that men exert more effort to inhibit the information of payoff levels and to use self-referencing to infer the strategies of partners with the goal of maximizing profit. Furthermore, men showed equivalent activation in the subgenual anterior cingulate cortex across payoff levels, whereas women showed a decreased activation with increasing payoff level – indicating decreased group bonding with higher risk in women. In conclusion, our results imply that women are more sensitive to social risk while trusting, which has implications for financial interactions, interpersonal relationships, and social involvement.

## Introduction

Trust is integral to relationships, cooperative behavior (Poppo et al., 2018), and a functioning society (Tov and Diener, 2009) and its dysfunction is a component of many mental disorders (King-Casas et al., 2008; Kéri et al., 2009; Sripada et al., 2009; Bell et al., 2019). Interpersonal trust is defined as the psychological state of a person (i.e., trustor) comprising the intention to accept vulnerability based upon positive expectations of behavior of another person (i.e., trustee) (Rousseau et al., 1998). The complexity and importance of interpersonal trust have motivated researchers to generate a wealth of experimental data over the years using standardized paradigms such as the trust game (Berg et al., 1995; Camerer, 2011).

In the standard version of the trust game, two players engage in a sequential, one-shot economic exchange. Player 1 (i.e., trustor) is given an endowment in monetary units (MU), of which the trustor can choose to send any amount (i.e., trust) or none (i.e., non-trust) to an anonymous player 2 (i.e., trustee). The MU sent by the trustor is then multiplied (e.g., doubled or tripled) by the experimenter. The trustee can choose to send back any amount of the received money (i.e., reciprocate) to the trustor or keep everything (i.e., betrayal). Although the subgame perfect Nash equilibrium for a rational player 1 – expecting that player 2 will not return any money – is to *not trust*, studies have shown that people typically send 50% of their initial endowment as player 1 and return about 37% of the money received as player 2 in one-shot trust game interactions (Johnson and Mislin, 2011).

A modification of the trust game – the experimental paradigm used in this study – is the binary multi-round trust game, where players alternate between the role of trustor and trustee with the same partner over multiple rounds and simply decide to *trust* or *not trust* and *reciprocate* or *betray* their partners’ trust. The binary multi-round trust game has a higher ecological validity compared to the standard trust game because trust relationships in real life are reciprocal and go through phases of trust building and maintenance (and/or trust violation and recovery).

An important interpersonal factor in trust relationships is gender, which describes the norms, roles, and identity of women and men (and other culture-specific genders). Gender norms and roles, such as women being communal and caring and men being agentic and independent, are internalized through socialization, also known as the social role theory (Eagly and Wood, 2012). These internalized norms are known to guide and influence all types of behavior on an unconscious as well as conscious level, such as when individuals are anxious about conforming to negative stereotypes about their gender (also known as “stereotype threat”). For example, one study found that stereotype threat led to increased loss- and risk aversion behavior in women but not men when faced with a financial decision (Carr and Steele, 2010). Furthermore, when a socialized individual is met with a situation in which she/he must choose whether to make her/himself vulnerable to another person or not – especially when little else is known about the other person – gender is a readily available piece of information that can be used to predict how the other person will behave. For example, we have strong beliefs about each gender’s trustworthiness (Slonim and Guillen, 2010; Zhao and Zhang, 2016), and the perceived trustworthiness of another person is known to be positively related to our decisions to trust (van’t Wout and Sanfey, 2008).

A recent meta-analysis of the one-shot trust game – encompassing 77 behavioral studies, 174 effect sizes, and 17,082 participants from 23 countries – found a robust effect (*g* = 0.22) of gender on trust, revealing that men send more money as player 1 than women (Van den Akker et al., 2018). In contrast, no overall effect of gender on trustworthiness was found: women and men were found to return about the same proportion of money received as player 2. One theory that has been proposed to explain the role that gender plays specifically in interpersonal trust is the parental investment theory (Trivers, 1972), which stems from Darwin’s sexual selection theory (Darwin, 1871). The parental investment theory posits that (based on biological differences in the “cost” of producing and nurturing offspring between females and males) females evolved through natural selection to avoid physical and social risks to ensure their reproductive potential, while males evolved to take risks to signal their health, status, and resources to potential mates (Trivers, 1972). Males’ reliance on using their resources to gain reproductive success, and females’ reliance on holding resources to invest in parenting, leads to the evolution of risk-seeking behavior in males and risk-aversive behavior in females. These adaptations may have been selected specifically for their implications on decision-making under uncertainty (Dreber and Hoffman, 2010). When it is considered that the gender roles and norms for women and men developed from the biological roles of females and males, the evolutionary and sociocultural theories combined explain that women are less trusting because they have more to lose from social interactions and need to be more sensitive to treachery.

In contrast, men are more trusting because it signals that they can afford to lose something and provides an opportunity to gain resources and become a more attractive mate. In line with this, men have been found to take more risks (Fischer and Hills, 2012; Apicella et al., 2017). Furthermore, studies investigating specifically the propensity to take risks in different social settings find that this type of risk-taking is associated with trust behavior in the one-shot trust game (Karlan, 2005; Ben-Ner and Halldorsson, 2010; Lönnqvist et al., 2015). Although evidence about gender differences in trust behavior exists, its underlying neuropsychological mechanisms are still obscure. Previous neuroimaging studies regarding gender differences in trust found stronger activation in the temporal-parietal junction in males, whereas stronger activation in the caudate in females (Lemmers-Jansen et al., 2017, 2019). However, these studies only investigated trust gender differences playing against a cooperative and an unfair partner and did not vary social risks.

While trust (and the trust game) originate in economics research, trust has increasingly become a topic of research in psychology and, more recently, social neuroscience. The findings from these different scientific fields, with their diverse methods and perspectives, are complementary. A neuropsychoeconomic model of trust was recently proposed that aims to integrate findings from the fields of behavioral economics, psychology, and social neuroscience (Krueger and Meyer-Lindenberg, 2019). According to this model, trust arises through the interplay of trust components (i.e., treachery, reward, uncertainty, strategy, and trustworthiness) – linked to psychological systems (i.e., motivation, affect, and cognition) – that engage key brain regions anchored in domain-general large-scale brain networks. The anticipation of reward (motivational system, reward network including striatum, and ventromedial prefrontal cortex) contrasted with the risk of treachery (affective system, salience network including ACC, and anterior insula) creates uncertainty, which is associated with vulnerability of trusting another person. To remove uncertainty, trustors can adopt a context-based strategy (cognitive system, central-executive network including lateral prefrontal cortex, and posterior parietal cortex) to recap personal benefits (i.e., economic rationality) and/or evaluate the relationship-based trustworthiness (social cognition, default-mode network including medial prefrontal cortex, and posterior cingulate cortex) to contribute to the relationship’s success (i.e., social rationality).

Interpersonal trust may evolve through repeated interactions from *calculus-based trust* (performing rational calculations of the costs and benefits of trust decisions driven mainly by the salience network), through *knowledge-based trust* (using knowledge about their context and the context of their partners to predict trustees’ behavior and advance their trust relationships, driven mainly by the cognitive control network to adopt a strategy or default-mode network to evaluate trustworthiness), to *identification-based trust* (developing a rewarding identification with trustees, driven mainly by reward processing/pair bonding network) (Krueger and Meyer-Lindenberg, 2019).

The goal of this study was to test the predictions of the parental investment theory – namely, that men trust more to maximize resources whereas women trust less due to higher sensitivity to betrayal – in an experimental setting. To achieve this goal, we re-analyzed our previously published data (Krueger et al., 2007). Unlike previous studies, we measured trust behavior with a binary multi-round trust game closely imitating trust relationships in real life where dyads of participants switched between the role of trustor and trustee after each round. Further, we let dyads play rounds of the trust game at different payoff levels, with increasing MU’s representing a higher social risk of betrayal not only in terms of material loss, but also with regard to the building and maintenance of the trust relationship over the course of the experiment. Our previously published study investigated the neural correlates of trust in partnership-building and maintenance stages in non-defector and defector groups, evidencing that conditional trust selectively activated the ventral tegmental area (reward system), whereas unconditional trust selectively activated the septal area (social attachment system) (Krueger et al., 2007). The present study focused on the gender differences of trust.

At the behavioral level, we hypothesized that dyads of both genders quickly develop a trust relationship from calculus-based over knowledge-based to identification-based trust due to the multi-round role-switching trust game format. However, we predicted that men trust more independently of payoff levels to maximize resources, whereas women trust less and adjust their trust based on payoff level to minimize their social risk. At the neural level, we predicted that to maximize resources, men utilize more brain regions associated with knowledge-based trust, implementing a context-based strategy to reap personal benefits (e.g., lateral prefrontal cortex) and evaluate the relationship-based trustworthiness to contribute to the relationship’s success (e.g., precuneus and temporal-parietal junction). Finally, we hypothesized that men develop identification-based trust, activating brain regions associated with reward/bonding processes independently of payoff levels, whereas women decrease their activation in those brain regions with higher payoff levels.

## Materials and Methods

### Participants

Forty-four (22 women and 22 men) healthy individuals were recruited via the subjects database of the National Institutes of Neurological Disorders and Stroke (NINDS) in Bethesda, Maryland, United States participated as same-gender dyads in an fMRI hyperscanning experiment (Montague et al., 2002) for financial compensation. We used same-gender dyads instead of opposite gender dyads to establish a valid start to test the interaction effect between social risk and gender. Dyads were matched by age (*M* ± *SD* = 28.4 ± 7.2 years, range = 21–51), education (17.3 ± 2.2 years, range = 12–23), and handedness (95.3 ± 8.7, range of 65–100, all right-handed) as assessed with the Edinburgh Handedness Inventory (Oldfield, 1971; Supplementary Table S1). The participants were native English speakers that had normal or corrected vision and were not taking any medication. Exclusion criteria were a history of medical, psychiatric, or neurological diagnoses and left-handedness. Prior to participating in the experiment, participants underwent a neurological examination as part of the screening procedure and provided informed consent in compliance with the standards of the NINDS Institutional Review Board. Note that the collected data from this study was used in a previous publication (Krueger et al., 2007).

### Procedure

The experiment consisted of three phases. During the *pre-scanning phase*, two strangers of the same gender – playing the trust game as a pair – were first instructed separately in different rooms. The participants were briefly allowed to see each other (via webcam) and then asked to rate the perceived closeness, partnership, and leadership of the other person based on their first impression. Participants were asked to rate the perceived closeness to their partner (not at all vs. very close; 0–10) and rank themselves in comparison to the other player by leadership (leader vs. follower; 0–10), and partnership (competitor vs. partner; 0–10). Digital pictures were taken of the participants’ neutral facial expression to be displayed to their partner on the screen during the trust game. Participants completed a practice run after being instructed about the experimental paradigm and private payment procedure at the end of the experiment.

**FIGURE 1:**
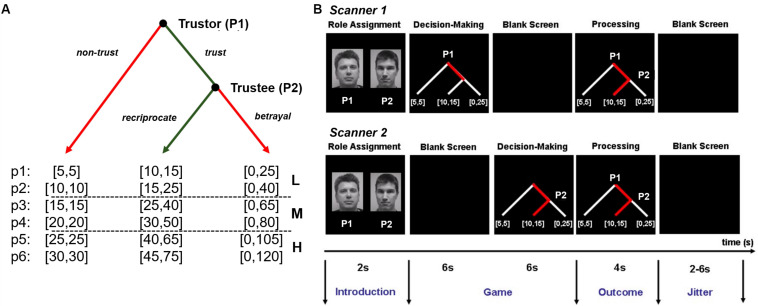
Experimental design. **(A)** Binary trust game. Partners made sequential decisions as player 1 (P1) and player 2 (P2) for payoffs in cents presented in a binary decision tree [c: (cP1, cP2)]. P1 can either move left (i.e., non-trust) or quit the game with a small payoff for P1 and P2 [e.g., (5, 5)] or right (i.e., trust) to continue the game. Next, P2 can move left (i.e., reciprocate) giving both players a higher payoff [e.g., (10, 15)] or right (i.e., betrayal) resulting in an even larger payoff for P1 and a payoff of zero for P1 [e.g., (0, 25)]. Payoffs (p1–p6) were split into three level: L (low: p1–p2), M (medium: p3–p4, and H (high: p5–p6). Numbers inside brackets indicate the specific payoff outcomes of the game trial. **(B)** Game trial. An introduction screen (2 s) informed partners about the role (P1 or P2) that they were playing. P1 made a decision (i.e., non-trust or trust) (within 6 s) while seeing the game tree and waited afterward (6 s) for P2’s decision while seeing a blank screen. After seeing a blank screen (6 s), P1 saw the game tree with P1’s decision and made a decision (i.e., reciprocate or betrayal) (within 6 s). If P1 had chosen not to trust P2, the game was over, and P2 saw P1’s decision (6 s). Partners saw the outcome of the game (4 s) followed by a blank screen with a jittered interstimulus interval (2–6 s). Adapted from Krueger et al. (2007), used with permission, Copyright (2007) National Academy of Sciences.

During the *scanning phase*, dyads completed the fMRI experiment that consisted of 36 sequential trust games (i.e., experimental condition) and 16 control games (i.e., control condition). They played six blocks (i.e., six games per block) of the trust game and four blocks (i.e., four games per block) of the control game (Supplementary Figure S1). Six different payoff levels (p1–p6) were used during the experiment and each level was only used once per trust game block (Figure 1A). Payoff levels were categorized into three types of *social risk* for analysis: low (p1–p2), medium (p3–p4), and high (p5–p6). For p2, p4, and p6, the initial endowment was tripled (but for p1, p3, and p5, to the tripled amount 5 MUs were added) if the trustor chose to trust, so it could be evenly split if the trustee chose to reciprocate (i.e., to return 50% of the received amount). The order of blocks, payoff levels, and display layouts were counterbalanced across games, dyads, and participants.

Participants alternated between playing as player 1 (P1) and player 2 (P2) every round: the role assignment appeared on the screen at the beginning of each round along with the decision tree displaying the specific payoff outcomes of the game round (Figure 1B). For each trial of the binary trust game, P1 was given the choice to *trust* or *not trust* the other player, risking all or nothing of her/his initial endowment. If P1 chose to not trust, then the decision was shown to P2, and the round ended with both players keeping their initial endowment (both players received the same small amount of MU at the beginning of each round). If P1 chose to trust, then the amount was sent over, and P2 was given the choice to *reciprocate* or *betray* (keep everything). The game then ended after both players were shown the outcome of P2’s decision. Participants were instructed to decide as quickly as possible: 100 MU were deducted from an individual player’s cumulative total earnings every time they failed to decide within 6 s.

The control game (identical to the trust game) was used to control for the monetary, sensorimotor, and decision-making aspects of the task, but the participants played alone and were only tasked with choosing the action that would lead to the highest monetary payoff (Supplementary Figure S2). Cumulative totals for the MU earned in the trust game were not displayed during the experiment.

During the *post-scanning phase*, participants were once again asked to rate the closeness, partnership, and leadership felt toward their partner. Further, participants rated certain aspects of their own gameplay in the fMRI experiment with one-item measures: cooperation (competitive vs. cooperative; 0–10), trustfulness (suspicious vs. trusting; 0–10), hemisphere (“left-brained”/intuitive vs. “right-brained”/analytic; 0–10), and game strategy (always used the same strategy vs. used many strategies; 0–10). Moreover, empathy was assessed with the *Z*-score of the Balanced Emotional Empathy Scale (BEES), a 30-item self-report of one’s tendency to experience other’s emotional experiences (Mehrabian and Epstein, 1972). Finally, participants received their accumulated earnings from the experiment (between 0 and 25 USD) in addition to a fixed compensation for participating in the fMRI experiment.

### Behavioral Data Analysis

Statistical analyses for the behavioral data was carried out with SPSS (Version 22.0.0.0, IBM Corporation 2013) applying a significance level of α < 0.05 (two-tailed). Standardized effect sizes were calculated, including Cohen’s *d* for *t*-tests and planned contrasts and η_p_^2^ (partial eta squared) for factors in analyses of variance (ANOVAs). The strength of effect sizes calculated using Cohen’s *d* was interpreted using the cutoffs > 0.20 for small, > 0.50 for medium, and > 0.80 for large, and for effect sizes calculated using η_p_^2^ > 0.01 for small, > 0.06 for medium, and > 0.14 for large (Cohen, 1988; Miles and Shevlin, 2001).

Decisions and reaction times for the trust and trustworthiness behavior were calculated for different levels of payoff and phases of the trust game. Mixed three-way 3 × 2 × 2 ANOVAs were conducted for those measures with Payoff (low, medium, and high) and Phase [building (run 1) vs. maintenance (run 2)] as within-subjects factors and Gender (men vs. women) as a between-subjects factor. The direction of significant effects involving Payoff were investigated *post hoc* with Bonferroni-corrected Helmert contrasts of low payoff vs. higher payoffs and moderate vs. high payoff.

Further, the effect of gender on perceived changes in the trust relationship over time was invested employing mixed two-way 2 × 2 ANOVAs for partner ratings (i.e., closeness, partnership, and leadership) with Time (pre- vs. post-game) as within-subjects factor and Gender (men vs. women) as between-subjects factor. Finally, the self-ratings of cooperation, trustfulness, hemispheric use, and variability in game strategy as well as the assessed personality characteristics of empathy were compared between men and women with independent samples *t*-tests to identify potential control variables for behavior in the trust game.

### Functional Image Acquisition and Preprocessing

Brain images were acquired on two 3-Tesla MRI whole-body scanners (General Electric) in the NMR Research Center at the National Institutes of Health. Head motion was restricted by using foam pads placed around the participant’s head. Anatomical images were acquired using T1-weighted MP-RAGE sequence (TR = 9.7 ms, TE = 4.0 ms, flip angle = 12°, field of view = 240 mm, thickness = 1.2 mm, in-plane resolution = 0.8594^∗^0.8594 mm^2^), and T2^∗^-weighted echo-planar images (EPI) optimized for BOLD contrast were collected (TR = 2 s, TE = 30 ms, flip angle = 90°, thickness = 6 mm, number of slices = 22, field of view = 240 mm, voxel dimensions = 3.75^∗^3.75^∗^6 mm). For each of the two functional runs, 291 volume images per run were taken parallel to AC–PC line. The first five volumes were discarded prior to analysis.

Image analyses were performed by using BrainVoyager QX (Version 20.6.2.3266 for Windows, Brain Innovation). Preprocessing steps included slice-time correction, linear trend removal, temporal high-pass filtering to remove low-frequency non-linear drifts of three or fewer cycles per time course, spatial smoothing (8 mm FWHM), and three-dimensional motion correction to detect and correct for small head movements by spatial alignment of all participants to the first volume by rigid body transformation. Estimated translation and rotation parameters were inspected and never exceeded 2 mm or 2°. To transform the functional data into Talairach space, the functional time series data of each subject were first co-registered with the subject’s three-dimensional anatomical data set and resampled to 3 mm × 3 mm × 3 mm isotropic voxels, resulting in a normalized four-dimensional volume time course data.

### Image Data Analysis

A general linear model (GLM) corrected for first-order serial correlation was applied (Friston et al., 1999). Random-effect analyses were performed on the multisubject level (group data: *n* = 44) and group-level (subgroup data: *n* = 22 men; *n* = 22 women) to explore brain regions that are associated with decisions to trust. For each participant, regressors were created based on individual decisions as P1 and P2 in the trust games (TG) [P1: trust_low_payoff (p1–p2), trust_medium_payoff (p3–p4), trust_high_payoff (p5–p6), non-trust; blank_screen; P2: reciprocate, betrayal, blank_screen; P1 and P2: introduction, outcome_reciprocate, outcome_betrayal] and control games (CG) (P1: choice, blank_screen; P2: choice, blank_screen; P1 and P2: introduction, outcome_P1, outcome_P2) over both functional runs [i.e., building (run1) and maintenance (run2) phase of the trust relationship]. The regression model consisted of a set of 36 (2 × 18) regressors (11 TG and 7 CG regressors per phase). Regressor time courses were adjusted for the hemodynamic response delay by convolution with a double-gamma hemodynamic response function (Büchel et al., 1998). Multiple regression analyses were performed independently for the time course of each individual voxel.

A mixed three-way 3 × 2 × 2 ANOVA for parameter estimates of each voxel was conducted with Payoff (low, medium, and high) and Phase [building (run 1) vs. maintenance (run 2)] as within-subjects factors and Gender (women vs. men) as a between-subjects factor. Brain activations for the decision phase were reported after correcting for multiple comparisons using a cluster-level statistical threshold – employing the cluster-level statistical threshold estimator plugin in BrainVoyager QX. The thresholded map (*p* < 0.005) was used for a whole-brain correction criterion, which is based off an estimate of the map’s spatial smoothness and on a Monte Carlo simulation (1,000 iterations). The minimum cluster size at a specified confidence level (α = 0.05) was then calculated (Forman et al., 1995; Goebel et al., 2006). The significant activation clusters were displayed in Talairach space on an average anatomical brain of all participants reversed left to right (i.e., radiological convention). Parameter estimates (mean weights) from activated brain regions were derived from the peak voxel activation and surrounding voxels encompassing 54 mm^3^.

## Results

### Behavioral Results

**FIGURE 2:**
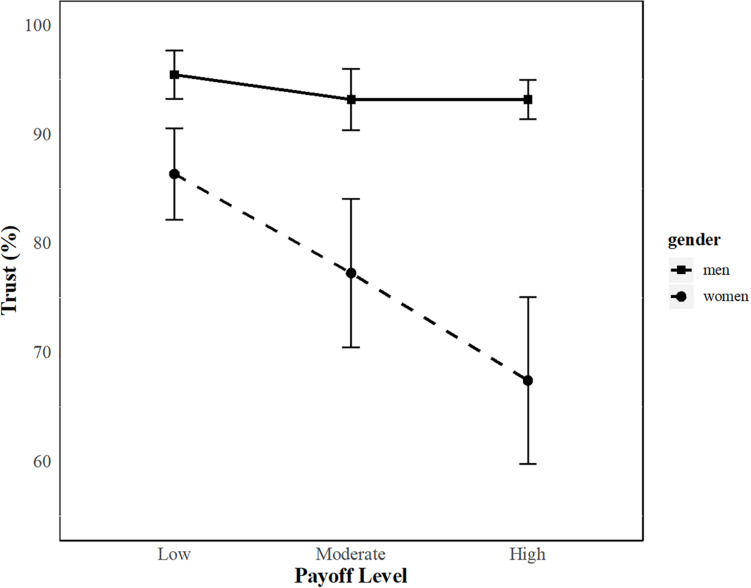
Trust as a function of gender and payoff level. Trust (mean ± standard error) decreased for women but stayed the same for men across all payoff levels.

Participants trusted their partner 85% of the time (*SD* = 21.4, range = 22.2–100). The ANOVA for trust behavior showed a significant main effect of Payoff [*F*_(2, 84)_ = 7.05, *p* = 0.001, η_p_^2^ = 0.14] (Supplementary Table S2), indicating that trust decreased with the level of payoff. Further, a significant main effect of Gender was found [*F*_(1, 42)_ = 7.42, *p* = 0.009, η_p_^2^ = 0.15], showing that men trusted more (∼94%) than women (∼77%) (Supplementary Table S11). Finally, a significant Payoff × Gender interaction effect (but no Payoff × Gender × Phase interaction) was demonstrated [*F*_(2, 84)_ = 5.17, *p* = 0.008, η_p_^2^ = 0.11], indicating that men showed the same levels of trust across all payoff levels, whereas women showed a decrease in trust with higher payoff levels independently of the phase for the trust relationship (Supplementary Figure S3). *Post hoc* tests revealed significant gender differences in trials with moderate payoff (*t*_42_ = 2.16, *p* = 0.04, *d* = 0.65) as well as high payoff (*t*_42_ = 3.29, *p* = 0.003, *d* = 0.99), but marginal significance in trials with low payoff (*t*_42_ = 1.91, *p* = 0.065, *d* = 0.58) (Figure 2). Men and women chose to reciprocate their partner’s trust about 85% of the time (*SD* = 25.8, range = 0–100). The ANOVA for trustworthiness behavior showed no significant main and interaction effects (Supplementary Table S3, Note that 6 women were subject to listwise exclusion because some women never trusted their partner for the high payoff level).

The ANOVA for response times showed only a significant Gender effect for trust [*F*_(1, 39)_ = 4.62, *p* = 0.038, η_p_^2^ = 0.11] but not for trustworthiness (Supplementary Tables S4, S5). Women were on average 113 ms faster than the men trusting their partner (women: *M* = 1359 ms, *SD* = 587; men: *M* = 1472 ms, *SD* = 736). Further, significant main effects of Phase were observed, indicating that participants (independently of gender) became faster to trust [*F*_(1, 39)_ = 7.23, *p* = 0.011, η_p_^2^ = 0.17] and reciprocate [*F*_(1, 36)_ = 6.40, *p* = 0.017, η_p_^2^ = 0.17] their partners from the building (run 1) to the maintenance (run 2) phase of the trust relationship.

The ANOVAs for participants’ changing beliefs about their partners over time showed no significant main effects of Gender on closeness [*F*_(1, 42)_ = 2.15, *p* = 0.150, η_p_^2^ = 0.05], partnership [*F*_(1, 42)_ = 0.87, *p* = 0.355, η_p_^2^ = 0.02], and leadership [*F*_(1, 42)_ = 0.12, *p* = 0.737, η_p_^2^ = 0.01] (Supplementary Tables S6–S8). However, significant main effects of Time were observed for closeness [*F*_(1, 42)_ = 14.5, *p* = 0.001, η_p_^2^ = 0.26] and partnership [*F*_(1, 42)_ = 4.82, *p* = 0.034, η_p_^2^ = 0.10] but not for leadership [*F*_(1, 42)_ = 2.90, *p* = 1.000, η_p_^2^ = 0.00]. Independently of gender, participants felt that they became closer to and developed a higher degree of partnership with their partner after playing the multi-round trust game.

Among the series of the assessed control and personality measures, women were using more strategies than men in the trust game (*t*_36_._0_ = 2.89, *p* = 0.006, *d* = 0.68) (Supplementary Table S9). Although women scored higher than men on empathy (*t*_31_._0_ = 2.86, *p* = 0.007, *d* = 0.65), the previously identified significant main effects of Payoff and Gender as well as the Payoff × Gender interaction effect remained significant after running a mixed three-way analysis of covariance (ANCOVA) for trust behavior with Payoff (low, medium, and high) and Phase (building vs. maintenance) as within-subjects factors, Gender (men vs. women) as a between-subjects factor, and Empathy as a covariate (Supplementary Table S10).

### Neuroimaging Results

**FIGURE 3:**
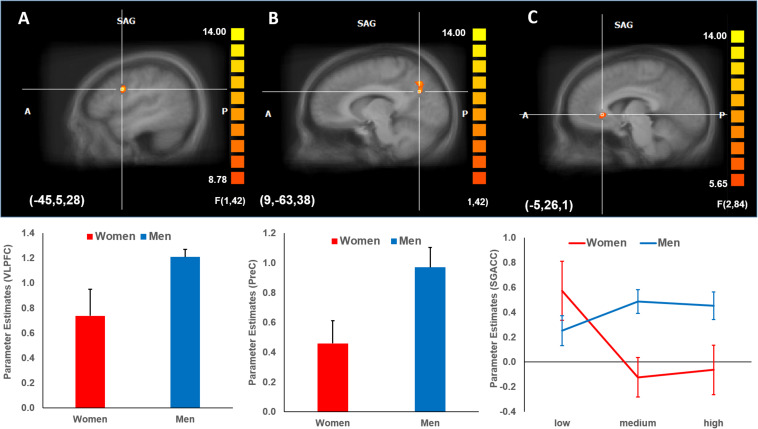
Brain activations (mean parameter estimates ± standard error) during decision phase corrected for multiple comparisons at the cluster level. **(A,B)** Gender effect. Men showed higher activation in the left inferior frontal gyrus (VLPFC, BA 44) and right precuneus (PreC, BA 7) compared to women (α < 0.05, *k* = 21). **(C)** Payoff x Gender interaction effect. Men activated consistently the left SgACC (BA 24) (α < 0.05, *k* = 14), whereas activation decreased for women across payoff levels. Parameter estimates (mean weights ± standard error) from activated brain regions were derived from the peak voxel activation and surrounding voxels encompassing 54 mm^3^. BA, Brodman area; VLPFC, ventrolateral prefrontal cortex; PreC, precuneus; SgACC, subgenual ACC; SAG, sagittal.

A mixed three-way 3 × 2 × 2 ANOVA was performed to identify brain activations during the decision phase of the trust game (corrected for multiple comparisons at the cluster level). A significant main effect of Gender was found, indicating a greater brain activation for men compared to women in the left inferior frontal gyrus (ventrolateral prefrontal cortex, VLPFC, BA 44; Tal −45, 5, 28) and right precuneus (PreC, BA 7; Tal 9, −63, 38) (α < 0.05, *k* = 21) (Figures 3A,B). Further, a significant interaction effect of Payoff × Gender (α < 0.05, *k* = 14) was found in which activation in the left subgenual anterior cingulate cortex (SgACC, BA 24; Tal −5, 26, 1) decreased with payoff levels in women compared to men (Figure 3C). Note that to avoid circularity (i.e., “double dipping”), no further statistical analyses were performed regarding this interaction effect (Kriegeskorte et al., 2009). No further brain activations for the remaining main and interactions effects were found.

## Discussion

The goal of this study was to investigate the underlying neuropsychological signatures of gender differences in trust by combining fMRI with a multi-round binary trust game that varied the social risk in the form of different payoff levels. On the behavioral level, we observed that women trusted less than men – decreasing their trust with the payoff level – but reciprocated the same. Further, we demonstrated on the neural level that men compared to women showed greater activation in the left VLPFC and right PreC, indicating a stronger recruitment of cognitive control and self-referencing strategies respectively in men for the goal of maximizing profit. Further, men exhibited similar activation across all payoff levels in the left SgACC – a region involved in group affiliation and reward processing – whereas women showed decreased activation with increasing payoff levels. Our results support the parental investment theory, which posits a gender-specific effect of social risk on trust behavior in women and higher trust among men to maximize resources.

In this study, both men and women trusted their partner about 85% of the time overall while playing a multi-round version of the binary trust game. Although similar in design to a previous study (Haselhuhn et al., 2015), our study was designed so that participants played repeatedly with the same partner while alternating roles as a trustor and trustee and, as such, were likely more motivated to develop a trust relationship. As a consequence, both men and women felt closer to their partners and reported a higher degree of partnership after completing the trust game. Further, they did not perceive a change in the degree of leadership shown by their partner, reflecting the reciprocal nature of trust relationships in which both partners are sometimes the trustor and sometimes the trustee. Moreover, to control for the possible confounding by discrimination against the opposite gender, participants were paired by same-gender, in which people may be involved in a parochial altruism situation (be altruistic to in-group members).

Despite the high amount of trust across genders, men trusted more than women overall independently of payoff levels, while women decreased their trust with increasing payoff levels as a measure of social risk. This remained the case after controlling for empathy, a personality trait on which women consistently score higher than men (Christov-Moore et al., 2014). Women were faster overall in deciding to trust and reported utilizing more strategies than men – pointing to adaptive behavior in the face of different levels of social risk.

Our results not only replicated the findings of a previous meta-analysis investigating gender differences in trust games (Van den Akker et al., 2018), but also provided empirical evidence for the parental investment theory. Women trusted less due to a higher sensitivity for social risk, supporting the assumption that gender norms for women and men are acquired through socialization “evolved” from a female biological imperative to avoid social risk and betrayal in relationships and the male motivation to maximize resources. These results are consistent with recent evidence showing that hormonal changes after competition predict sex-differentiated decision-making, i.e., women make more conservative decisions and men more riskier decisions after experiencing a competitive social context (Alacreu-Crespo et al., 2019).

Mirroring our findings on the behavioral level that men trust more than women, men showed greater activation in the left VLPFC and right PreC at the neural level.

On the one hand, the VLPFC has been linked to enhancing goal-directed behavior and improving long-term outcomes during trust decisions (Krueger and Meyer-Lindenberg, 2019). Although a broad array of functions are associated with the VLPFC – language processing (Wagner et al., 2014), mental imagery (Kleider-Offutt et al., 2019), planning (Fincham et al., 2002), selective bias of behaviorally relevant information (Blackwood et al., 2000), and selection among competing information to guide a response (Thompson-Schill et al., 1998) – former findings emphasize the role of the VLPFC in cognitive control and inhibition (Bereczkei et al., 2015). For example, a previous study revealed that control of distrust and individual differences in change of distrust are linked with left VLPFC activity, reflecting an increased engagement of cognitive control in individuals who tend to change their distrust evaluations (Filkowski et al., 2016).

In our study, increased activation of the left VLPFC was found in men compared to women, suggesting that men engaged more cognitive control while trusting for the purpose of maximizing the reward. The VLPFC may play a crucial role in the evaluation of signals associated with the others’ social behavior and show elevated activity when the trustor faces a moderate or high risk of betrayal. Increased recruitment of the left VLPFC in men may indicate that they are more willing to pursue larger rewards by trust despite the increased social risk and uncertainty-related costs of doing so (as reflected by the insensitivity to payoff levels). To maximize their profit, men may selectively inhibit information about social risk and maintain a positively biased expectation of their partner’s reciprocity. This interpretation is supported by the questionnaire data from the post-scanning phase of the experiment: men reported using fewer strategies in the trust game to fulfill their economic goals. In addition, men showed longer response times than women across payoff levels, indicating probably that additional time was spent on the inhibition process.

On the other hand, the right PreC (stronger engaged by men than women) is a key region of the default-mode network (Cavanna and Trimble, 2006), which has been implicated in social cognition processes such as mentalizing (Zaki and Ochsner, 2012; Suzuki et al., 2019), tracking social distance (Tavares et al., 2015), and processing social identity (Volz et al., 2009). Previous trust studies have identified the involvement of the PreC in trust decisions (Emonds et al., 2014), and its activation is positively associated with an individuals’ level of trust as the result of perspective-taking (Prochazkova et al., 2018). More generally, the PreC may be involved in the evaluation of positive social interactions such as other’s benevolence and trustworthiness (Fett et al., 2014; McAdams et al., 2015). The greater PreC response observed in men in our study suggest that the PreC may also play a role in strategic trust decisions. In a multi-round trust game with the same partner and role-switching, men may have been more motivated to always trust as trustors to signal their trustworthiness to maximize profits. Greater PreC responses are consistent with males’ enhanced tendency to use self-referencing to infer the strategies of partners in the repeated trust game (Lambert et al., 2017). This confirms a prior study suggesting that PreC activations are linked to attempts to understand the responsiveness of others (Sakaiya et al., 2013).

Finally, the SgACC was differently activated for genders depending on the payoff level. A recent review on moral motivation examined the role of the SgACC in moral choices, with stronger activation associated with higher empathic concern, more donations, enhanced aversion to third-party harm, feelings of guilt, and group affiliation (Zahn et al., 2020). This review posited that the SgACC may represent attachment-related values of social outcomes. This suggestion is in line with the finding that the SgACC is activated both in response to personal and vicarious reward as identified by a meta-analysis (Morelli et al., 2015) as well as with the finding of another meta-analysis that the SgACC is preferentially recruited during altruistic giving (Cutler and Campbell-Meiklejohn, 2019).

The SgACC activation pattern in our study – decreased activation with increased social risk in women and no activation differences across all payoff levels in men – suggests that women adapt their strategy toward partners when the social risk increases. Both women and men could quickly form identification-based trust in the multi-round role-switching trust game and built up partnership in their pair. However, women were more sensitive to the social risk of betrayal. When the social risk became salient, women chose to detach themselves from the partnership and decrease their concern for their partner. In contrast, men were more focused on implementing a profit-maximizing strategy and gaining rewards, pursuing this target irrespective of payoff level. Overall, the described pattern was captured by SgACC activation, probably representing decreased pair bonding among women in moderate and high-risk contexts.

Unlike the behavioral results, we failed to find a main effect of payoff at the neural level. This may be due to a modest degree of repetition for each payoff level (12 trials for each level), which may have resulted in a lack of power to detect a subtle effect of risk on brain activity. Another possibility is that the social risk effect was masked by gender. As shown before, men were more focused on rewards irrespective of payoff level as evidenced by both behavioral and neural results. Only women distinguished between low and moderate/high risk levels; therefore, the risk effect might have been mitigated by this diversity.

Some limitations of our study must be considered. First, participants only played the game with a partner of the same gender. Whether playing with the opposite gender would show the same gender effect needs further investigation. As our study was the first to test the interaction effect between social risk and gender, it was more appropriate to use same-gender and same-race dyads to establish a valid start for this line of research. Future studies should investigate whether the observed interaction between social risk and gender can also be observed in mixed-gender dyads as well as in multiple, one-shot trust games with different partners (e.g., in-group vs. out-group members, same vs. different sexual orientation).

Second, our current neural findings should be considered with caution due to the lowered significance threshold for the whole-brain analysis (uncorrected *p* < 0.005 before correcting for multiple comparisons at the cluster level). However, the activation pattern replicated previous trust studies, which provides evidence the reliability of the current results (Emonds et al., 2014; Prochazkova et al., 2018). Future studies may shed further light on the neural mechanisms underlying gender differences by including large samples to effectively detect the subtle gender differences in associated brain regions.

## Conclusion

Despite those limitations, our study is the first to manipulate social risk in the trust game to investigate its moderating role in the observed gender differences in trust behavior using functional neuroimaging and show neural-psychological evidence that there is a tradeoff between social risk and partnership in women. Women trust less than men, and this effect is stronger when the social risk increased for the trust decision. We also demonstrated that men are more determined to adopt a constant strategy to maximize resources and to stick to this goal, as reflected by the greater activation in the VLPFC and PreC. In contrast, women were more sensitive to the social risk of betrayal, as indicated by the decreased SgACC activation when social risk increased. These results provide support for the prediction made by the parental investment theory. Our findings that women are more sensitive to social risk while trusting have implications for various aspect of social life such as financial interactions, interpersonal relationships, and social structures.

## Data Availability Statement

The datasets generated for this study are available on request to the corresponding author.

## Ethics Statement

The studies involving human participants were reviewed and approved by the NINDS Institutional Review Board. The patients/participants provided their written informed consent to participate in this study.

## Author Contributions

JG and FK designed the study. FK collected the data. AH, SS, and FK analyzed the data. YW, AH, and FK prepared the first draft of the manuscript. All authors contributed to the final version.

## Conflict of Interest

The authors declare that the research was conducted in the absence of any commercial or financial relationships that could be construed as a potential conflict of interest.
