# Supplementary material for: Neural Signatures of Gender Differences in Interpersonal Trust
Source: Front Hum Neurosci. 2020 Jun 16;14:225. doi: 10.3389/fnhum.2020.00225 (PMC7309600; doi:10.3389/fnhum.2020.00225)
Supplement: Supplementary file 1 [file Data_Sheet_1.docx]

**Supplementary Material**

**1. Supplementary Tables**

**Table S1.** Descriptive statistics (mean ± standard error of mean) of demographic variables

|  | **Men** | **Women** | ***t*** | ***p*** |
| --- | --- | --- | --- | --- |
| **N** | 22 | 22 |  |  |
| **Age** | 27.2 ± 1.16 | 29.6 ± 1.83 | 1.12 | 0.271 |
| **Education** | 17.2 ± 0.50 | 17.5 ± 0.45 | 0.41 | 0.686 |
| **Handedness** | 93.9 ± 2.25 | 96.8 ± 1.35 | 1.13 | 0.266 |

**Table S2**. Three-way ANOVA for trust as a function of Phase, Payoff, and Gender

| **Predictor** | **dfNum** | **dfDen** | **SSNum** | **SSDen** | ***F*** | ***p*** | ***ηp²*** |
| --- | --- | --- | --- | --- | --- | --- | --- |
| **Gender** | 1 | 42 | 3249 | 166679 | 7.93 | 0.007** | 0.16 |
| **Payoff** | 2 | 84 | 4958 | 27878 | 7.47 | 0.001** | 0.15 |
| **Phase** | 1 | 84 | 509 | 25025 | 0.86 | 0.361 | 0.02 |
| **Payoff*Gender** | 2 | 84 | 3089 | 27878 | 4.65 | 0.012* | 0.10 |
| **Phase*Gender** | 1 | 84 | 947 | 25025 | 1.59 | 0.214 | 0.04 |
| **Payoff*Phase** | 2 | 84 | 1423 | 21717 | 2.75 | 0.070 | 0.06 |
| **Gender*Payoff*Phase** | 2 | 84 | 934 | 21717 | 1.81 | 0.170 | 0.04 |

Note. *dfNum* = numerator degrees of freedom; *dfDen* = denominator degrees of freedom; *SSNum* = numerator sum of squares; *SSDen* = numerator sum of squares numerator; *ηp²* = effect size estimator partial eta-square. Payoff level „low“ and „men“ are the reference group.

* *p* < 0.05, ** *p* < 0.01, *** *p* < 0.001

**Table S3.** Three-way ANOVA for trustworthiness as a function of Phase, Payoff, and Gender

| **Predictor** | **dfNum** | **dfDen** | **SSNum** | **SSDen** | ***F*** | ***p*** | ***η_p_²*** |
| --- | --- | --- | --- | --- | --- | --- | --- |
| **Gender** | 1 | 36 | 61.3 | 6246 | 0.35 | 0.556 | 0.01 |
| **Payoff** | 2 | 72 | 1955 | 2849 | 2.43 | 0.095 | 0.06 |
| **Phase** | 1 | 72 | 778 | 11956 | 2.34 | 0.135 | 0.06 |
| **Payoff*Gender** | 2 | 72 | 2496 | 2849 | 0.05 | 0.051 | 0.08 |
| **Phase*Gender** | 1 | 72 | 929 | 11956 | 2.80 | 0.103 | 0.07 |
| **Payoff*Phase** | 2 | 72 | 484 | 13748 | 1.27 | 0.288 | 0.03 |
| **Gender*Payoff*Phase** | 2 | 72 | 69.8 | 13748 | 0.18 | 0.833 | 0.01 |

*Note.* The data of *n*=6 women was subject to listwise exclusion because of partial missing data as trustee (i.e., some women were never trusted by their partner, and as such were not able to make decisions as trustee). *dfNum* = numerator degrees of freedom; *dfDen* = denominator degrees of freedom*; SSNum* = numerator sum of squares; *SSDen* = numerator sum of squares numerator; η_p_² = effect size estimator partial eta-square. Payoff level „low“ and „men“ are the reference group.

* *p* < 0.05, ** *p* < 0.01, *** *p* < 0.001

**Table S4.** Three-way ANOVA for response times for decisions to trust as a function of phase, payoff level, and gender

| **Predictor** | **dfNum** | **dfDen** | **SSNum** | **SSDen** | ***F*** | ***p*** | ***η_p_²*** |
| --- | --- | --- | --- | --- | --- | --- | --- |
| **Gender** | 1 | 39 | 2153692 | 16788386 | 4.62 | 0.038* | 0.11 |
| **Payoff** | 1.41 | 50.8 | 328286 | 47440331 | 0.25 | 0.702 | 0.01 |
| **Phase** | 1.00 | 36.0 | 6014887 | 29934443 | 7.23 | 0.011* | 0.17 |
| **Payoff*Gender** | 1.41 | 50.8 | 1484769 | 47440331 | 1.13 | 0.315 | 0.03 |
| **Phase*Gender** | 1.00 | 36.0 | 195007 | 29934443 | 0.24 | 0.631 | 0.01 |
| **Payoff*Phase** | 1.23 | 44.3 | 883367 | 52185732 | 0.61 | 0.472 | 0.02 |
| **Gender*Payoff*Phase** | 1.23 | 44.3 | 907036 | 52185732 | 0.63 | 0.465 | 0.02 |

*Note.* The data of *n*=6 women was subject to listwise exclusion because of partial missing data as player 1 (i.e., some women never trusted their partner for specific levels of payoff or phases). *dfNum* = numerator degrees of freedom; *dfDen* = denominator degrees of freedom*; SSNum* = numerator sum of squares; *SSDen* = numerator sum of squares numerator; *η*_p_² = effect size estimator partial eta-square. Payoff level „low“ and „men“ are the reference group.

* *p* < 0.05, ** *p* < 0.01, *** *p* < 0.001

**Table S5.** Three-way ANOVA for response times for decisions to reciprocate as a function of phase, payoff level, and gender

| **Predictor** | **dfNum** | **dfDen** | **SSNum** | **SSDen** | ***F*** | ***p*** | ***η_p_²*** |
| --- | --- | --- | --- | --- | --- | --- | --- |
| **Gender** | 1 | 36 | 220232 | 6455144 | 1.23 | 0.275 | 0.03 |
| **Payoff** | 1.33 | 41.2 | 697385 | 39373270 | 0.55 | 0.511 | 0.02 |
| **Phase** | 1.00 | 31.0 | 5041740 | 24409408 | 6.40 | 0.017* | 0.17 |
| **Payoff*Gender** | 1.33 | 41.2 | 574838 | 39373270 | 0.45 | 0.560 | 0.01 |
| **Phase*Gender** | 1.00 | 31.0 | 776999 | 24409408 | 0.99 | 0.328 | 0.03 |
| **Payoff*Phase** | 1.31 | 40.6 | 1845811 | 45964439 | 1.25 | 0.284 | 0.04 |
| **Gender*Payoff*Phase** | 1.31 | 40.6 | 1093713 | 45964439 | 0.74 | 0.430 | 0.023 |

*Note.* The data of *n*=8 women and *n*=3 men was subject to listwise exclusion because of partial missing data (i.e., no recorded decisions to reciprocate for specific levels of payoff or phases because of non-trust of the partner). *dfNum* = numerator degrees of freedom; *dfDen* = denominator degrees of freedom*; SS_Num_* = numerator sum of squares; *SSDen* = numerator sum of squares numerator; *η*_p_² = effect size estimator partial eta-square. Payoff level „low“ and „men“ are the reference group.

* *p* < 0.05, ** *p* < 0.01, *** *p* < 0.001

**Table S6.** Two-way ANOVA for closeness rating of partner as a function of time and gender

| **Predictor** | **dfNum** | **dfDen** | **SSNum** | **SSDen** | ***F*** | ***p*** | ***ηp²*** |
| --- | --- | --- | --- | --- | --- | --- | --- |
| **Gender** | 1 | 42 | 12.6 | 245 | 2.15 | 0.150 | 0.05 |
| **Timepoint** | 1 | 42 | 78.3 | 226 | 14.5 | 0.001*** | 0.26 |
| **Gender*Timepoint** | 1 | 42 | 0.011 | 226 | 0.002 | 0.964 | 0.01 |

*Note. dfNum* = numerator degrees of freedom; *dfDen* = denominator degrees of freedom*; SSNum* = numerator sum of squares; *SSDen* = numerator sum of squares numerator; *η*p² = effect size estimator partial eta-square. Payoff level „low“ and „men“ are the reference group.

* *p* < 0.05, ** *p* < 0.01, *** *p* < 0.001

**Table S7.** Two-way ANOVA for partnership rating of partner as a function of time and gender

| **Predictor** | **dfNum** | **dfDen** | **SSNum** | **SSDen** | ***F*** | ***p*** | ***η_p_²*** |
| --- | --- | --- | --- | --- | --- | --- | --- |
| **Gender** | 1 | 42 | 2.75 | 132 | 0.87 | 0.355 | 0.02 |
| **Timepoint** | 1 | 42 | 24.0 | 210 | 4.82 | 0.034* | 0.10 |
| **Gender*Timepoint** | 1 | 42 | 2.23 | 53.3 | 0.45 | 0.508 | 0.01 |

*Note. dfNum* = numerator degrees of freedom; *dfDen* = denominator degrees of freedom*; SSNum* = numerator sum of squares; *SSDen* = numerator sum of squares numerator; *η*_p_² = effect size estimator partial eta-square. Payoff level „low“ and „men“ are the reference group.

* *p* < 0.05, ** *p* < 0.01, *** *p* < 0.001

**Table S8.** Two-way ANOVA for leadership rating of partner as a function of time and gender

| **Predictor** | **dfNum** | **dfDen** | **SSNum** | **SSDen** | ***F*** | ***p*** | ***η_p_²*** |
| --- | --- | --- | --- | --- | --- | --- | --- |
| **Gender** | 1 | 42 | 0.091 | 33.2 | 0.12 | 0.737 | 0.01 |
| **Timepoint** | 1 | 42 | 3.68 | 53.3 | 2.90 | 0.096 | 0.06 |
| **Gender*Timepoint** | 1 | 42 | 0.000 | 53.3 | 0.000 | 1.000 | 0.00 |

*Note. dfNum* = numerator degrees of freedom; *dfDen* = denominator degrees of freedom*; SSNum* = numerator sum of squares; *SSDen* = numerator sum of squares numerator; *η*_p_² = effect size estimator partial eta-square. Payoff level „low“ and „men“ are the reference group.

* *p* < 0.05, ** *p* < 0.01, *** *p* < 0.001

**Table S9.** Descriptive statistics (mean ± standard error) of demographic and control variables as well as earnings as a function of gender

|  | **Men** | **Women** | ***t*** | ***p*** | **Mean difference [CI]** | ***d*** |
| --- | --- | --- | --- | --- | --- | --- |
| **N** | 22 | 22 |  |  |  |  |
| **Empathy** | -0.22 ± 0.17 | 0.61 ± 0.24 | 2.86 | 0.007** | 0.83 [0.26, 1.38] | 0.75 |
| **Cooperation** | 7.95 ± 0.45 | 8.59 ± 0.53 | 0.91 | 0.366 | 0.64 [-0.77, 2.00] | 0.28 |
| **Trustfulness** | 7.45 ± 0.49 | 7.41 ± 0.62 | -0.06 | 0.954 | -.045 [-1.57, 1.46] | -0.02 |
| **Hemisphere** | 6.68 ± 0.46 | 5.50 ± 0.53 | -1.68 | 0.100 | -1.18 [-2.48, 0.13] | 0.48 |
| **Strategy** | 6.68 ± 0.59 | 8.73 ± 0.38 | 2.89 | 0.006** | 2.05 [0.57, 3.48] | 0.87 |

*Note.* CI = bootstrapped 95% confidence interval for mean difference; *d* = Cohen’s d for effect sizes.

* *p* < 0.05, ** *p* < 0.01, *** *p* < 0.001

**Table S10.** Three-way ANCOVA for trust as a function of Phase, Payoff, and Gender with the Empathy as covariate

| **Predictor** | **dfNum** | **dfDen** | **SSNum** | **SSDen** | ***F*** | ***p*** | ***η_p_²*** |
| --- | --- | --- | --- | --- | --- | --- | --- |
| **Gender** | 1 | 41 | 2.04 | 15.1 | 5.54 | 0.023* | 0.12 |
| **Empathy** | 1 | 41 | .0028 | 15.1 | 0.08 | 0.784 | 0.01 |
| **Payoff** | 2 | 82 | 4.33 | 24.6 | 7.21 | 0.001** | 0.15 |
| **Phase** | 1 | 41 | 0.16 | 23.3 | 0.28 | 0.598 | 0.08 |
| **Payoff*Gender** | 2 | 82 | 3.139 | 24.6 | 5.23 | 0.007** | 0.11 |
| **Phase*Gender** | 1 | 41 | 0.46 | 23.3 | 0.81 | 0.374 | 0.02 |
| **Payoff*Empathy** | 2 | 82 | 0.187 | 24.6 | 0.31 | 0.730 | 0.09 |
| **Phase*Empathy** | 1 | 41 | 0.48 | 23.3 | 0.85 | 0.362 | 0.02 |
| **Payoff*Phase** | 2 | 82 | 0.85 | 18.9 | 1.84 | 0.166 | 0.04 |
| **Payoff*Phase*Empathy** | 2 | 82 | 0.32 | 18.9 | 0.69 | 0.506 | 0.02 |
| **Payoff*Phase*Gender** | 2 | 82 | 0.56 | 18.9 | 1.21 | 0.304 | 0.03 |

*Note. dfNum* = numerator degrees of freedom; *dfDen* = denominator degrees of freedom*; SSNum* = numerator sum of squares; *SSDen* = numerator sum of squares numerator; η_p_² = effect size estimator partial etasquare. Payoff level „low“ and „men“ are the reference group.

* *p* < 0.05, ** *p* < 0.01, *** *p* < 0.001.

**Table S11.** Descriptive statistics (mean ± standard error) of trust and trustworthiness as a function of gender

|  | **Men** | **Women** |
| --- | --- | --- |
| **Payoff** | **Trust (%)** | |
| **Low** | 95.45 ± 2.24 | 88.36 ± 4.19 |
| **Moderate** | 93.18 ± 2.82 | 77.27 ± 6.81 |
| **High payoff** | 93.18 ± 1.79 | 67.42 ± 7.64 |
|  | **Trustworthiness (%)** | |
| **Low** | 96.97 ± 1.40 | 82.95 ± 6.91 |
| **Moderate** | 96.29 ± 2.18 | 75.15 ± 8.30 |
| **High** | 83.63 ± 6.02 | 82.63 ± 7.40 |

**2 Supplementary Figures**

**Figure S1. Trust game design**. The experiment was split into two stages (building [run1] and maintenance [run2]), each including 18 trust games and 8 control games. Each stage lasted about 12 min and consisted of three blocks of trust games (six games per block) and two blocks of control games (four games per block).

**Figure S2. Control game.** Control game decision tree for trial played in the role of a trustor (a) and trustee (b). Partners were asked to make sequential decisions as player 1 (P1) and player 2 (P2) in a binary decision game tree by either pressing the left (move left) or the right (move right) response button. Players did not interact with one another and merely had to choose between lower and higher payoff in cents [c: (cP1,cP2)]. P1 can choose the payoff by either moving left or right (along the solid line) in the decision tree (e.g., 0 or 5 cents), and P2 can choose the payoff by either moving left or right (along the dashed line) in the decision tree (e.g., 25 or 15 cents). For the control games, the same payoffs (p1-p6) were used as in the trust games.

**Figure S3. Trust as a function of gender, payoff, and phase.**

Trust (mean ± standard error) decreased for women but stayed the same for men across all payoff levels independently of the phase for the trust relationship (building [run 1] vs. maintenance [run 2]).
