# Supplementary figures and images for: Neural Signatures of Gender Differences in Interpersonal Trust
Source: Front Hum Neurosci. 2020 Jun 16;14:225. doi: 10.3389/fnhum.2020.00225 (PMC7309600; doi:10.3389/fnhum.2020.00225)

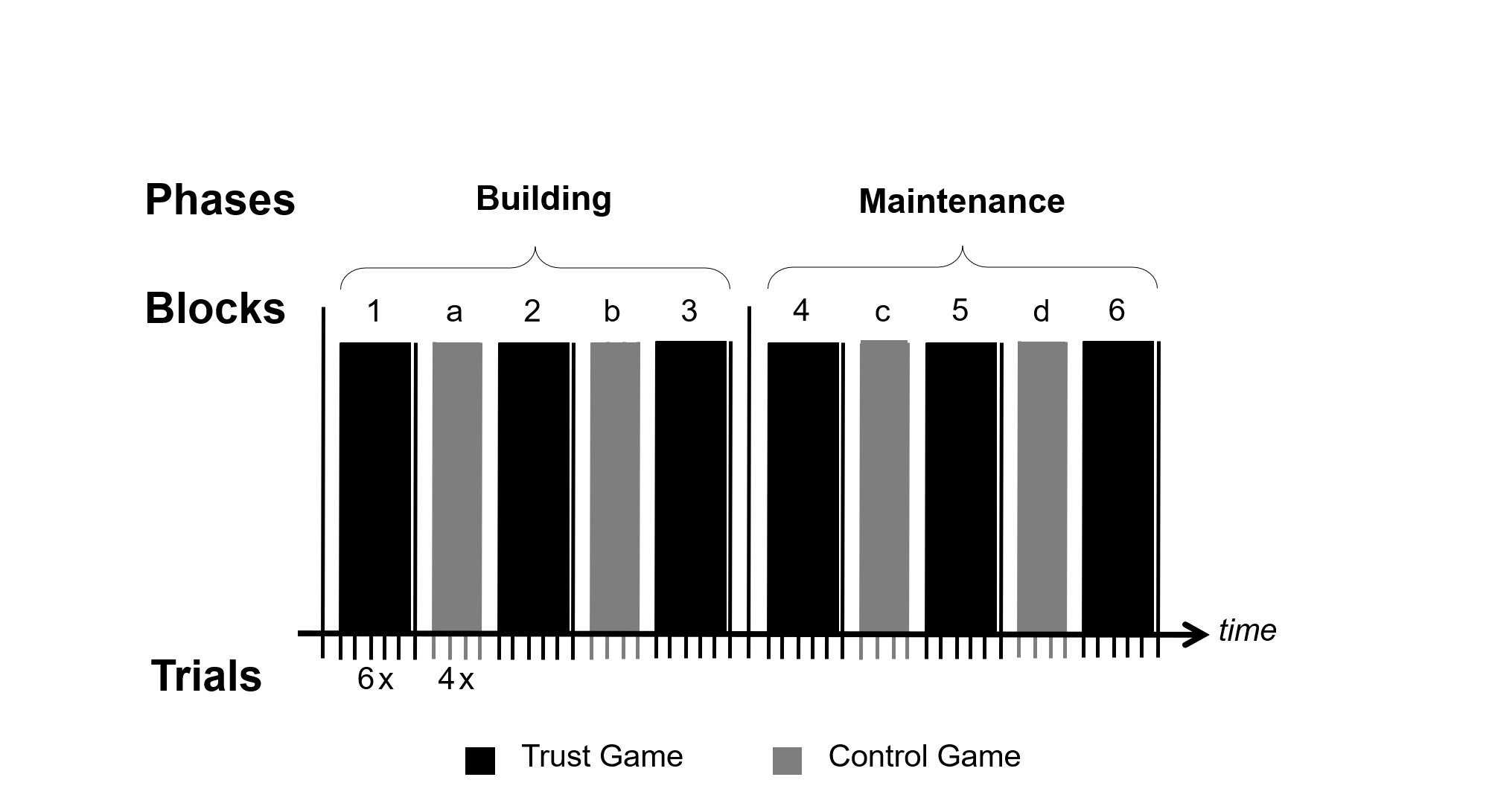

Supplement: Supplementary file 2 [file Image_1.TIF]

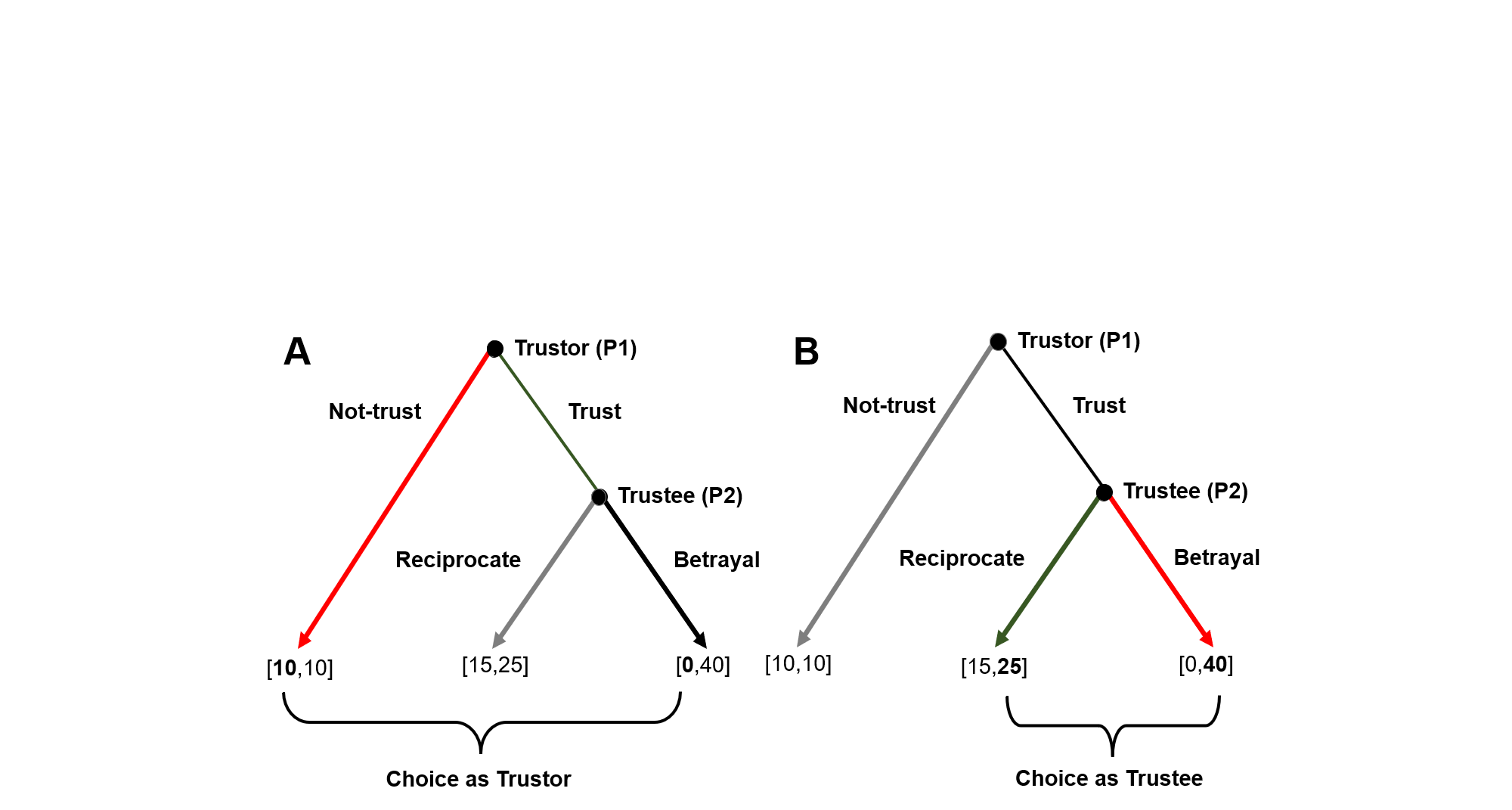

Supplement: Supplementary file 3 [file Image_2.TIF]

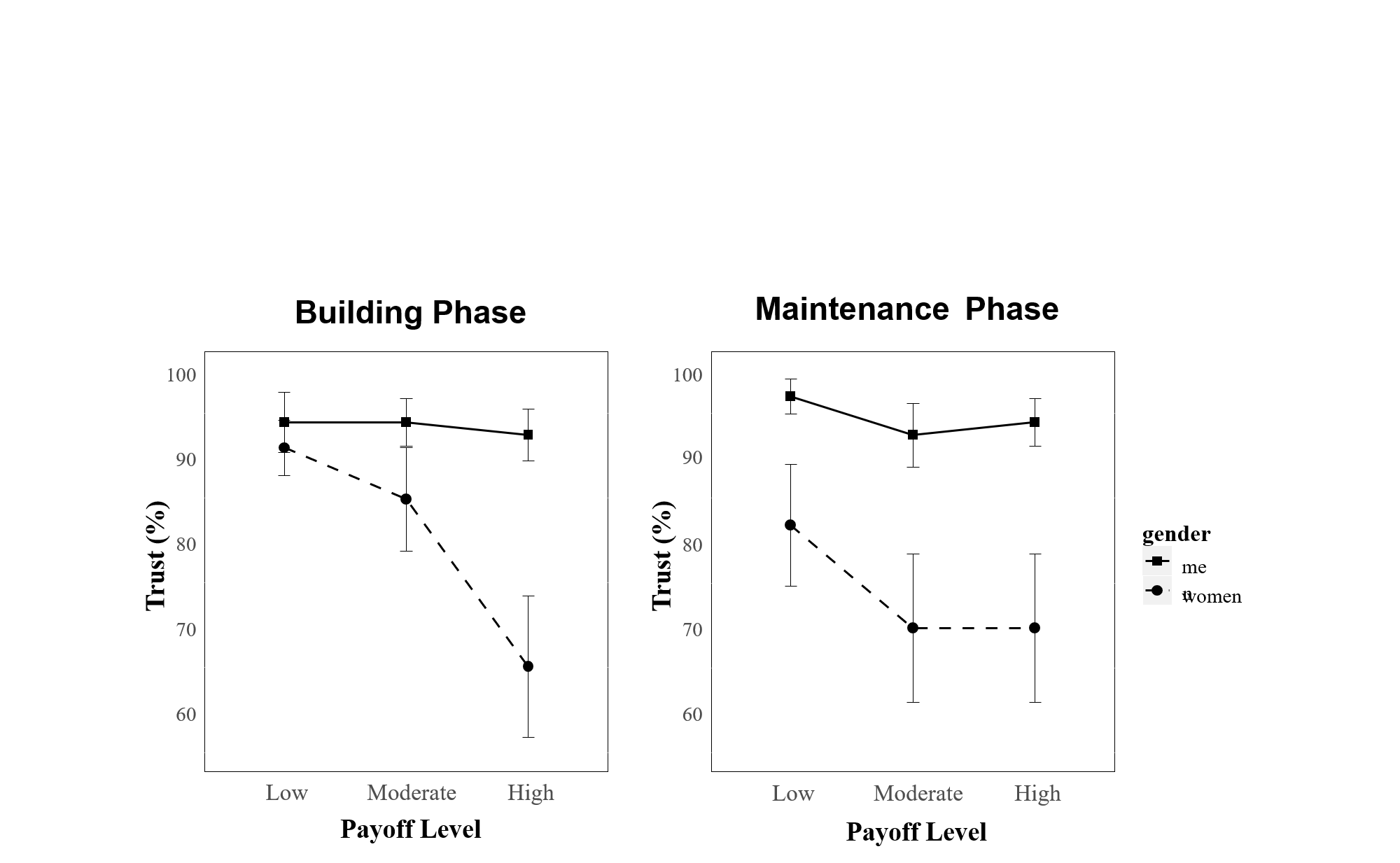

Supplement: Supplementary file 4 [file Image_3.TIF]
